# Analysis of risk factors and construction of a prediction model for short stature in children

**DOI:** 10.3389/fped.2022.1006011

**Published:** 2022-12-06

**Authors:** Shaojun Huang, Zhiqi Chen, Rongping Chen, Zhen Zhang, Jia Sun, Hong Chen

**Affiliations:** Department of Endocrinology, Zhujiang Hospital, Southern Medical University, Guangzhou, China

**Keywords:** short stature, children, risk factors, nomogram prediction model, risk classification system

## Abstract

**Background:**

Short stature in children is an important global health issue. This study aimed to analyze the risk factors associated with short stature and to construct a clinical prediction model and risk classification system for short stature.

**Methods:**

This cross-sectional study included 12,504 children aged 6–14 years of age from 13 primary and secondary schools in Pingshan District, Shenzhen. A physical examination was performed to measure the height and weight of the children. Questionnaires were used to obtain information about children and their parents, including sex, age, family environment, social environment, maternal conditions during pregnancy, birth and feeding, and lifestyle. The age confounding variable was adjusted through a 1 : 1 propensity score matching (PSM) analysis and 1,076 children were selected for risk factor analysis.

**Results:**

The prevalence of short stature in children aged 6–14 years was 4.3% in the Pingshan District, Shenzhen. The multivariate logistic regression model showed that the influencing factors for short stature were father's height, mother's height, annual family income, father's level of education and parents’ concern for their children's height in the future (*P* < 0.05). Based on the short stature multivariate logistic regression model, a short stature nomogram prediction model was constructed. The area under the ROC curve (AUC) was 0.748, indicating a good degree of discrimination of the nomogram. According to the calibration curve, the Hosmer–Lemesio test value was 0.917, and the model was considered to be accurate. Based on a risk classification system derived from the nomogram prediction model, the total score of the nomogram was 127.5, which is considered the cutoff point to divides all children into low-risk and high-risk groups.

**Conclusion:**

This study analyzed the risk factors for short stature in children and constructed a nomogram prediction model and a risk classification system based on these risk factors, as well as providing short stature screening and assessment individually.

## Introduction

Short stature has gradually become an important global health issue. Short stature is defined as a condition in which body height falls within in the third percentile or falls more than two standard deviations (SD) below the mean for the same sex and age group (1).

In recent years, many studies have been conducted examining children's heights in different locations and regions (2–4). These studies showed that the total population of individuals with short stature is still large worldwide, especially in developing countries (5, 6). Children with short stature lack confidence and have different degrees of adjustment, cognitive, and self-consciousness disorders (7, 8). They are under enormous psychological pressure, which affects their personal social skills, and working environment. Short stature in childhood, if not intervened, can increase short stature in adulthood and increases the risk of mental illness, abnormal lipid metabolism, diabetes, and cardiovascular events (9–13). Therefore, based on the concerning situation of the high prevalence of short stature and the significant consequences, we should actively investigate the epidemiological characteristics and current situation of short stature to provide practical evidence for the prevention and treatment of short stature.

Short stature in children is affected by both genetic and non-genetic factors. Heredity is a vital factor in height that plays a dominant position, which can contribute to 80% or more of height variation, indicating that height is usually a multigene regulation process (14). The influence of non-genetic factors on short stature is also crucial, including children's obesity, social environment, family environment, and lifestyle (15, 16). In a Swedish study, analysis using longitudinal growth data (*n* = 1,901) with an age 3.5 of 8 years showed that an increase in height during adolescence was negatively correlated with the peak value of the body mass index (BMI) in childhood (17). In a 15-year Swiss study, it was found that short stature in children can cause serious psychological problems and is directly related to pressure from the family environment (18). The growth of a child's height involves several factors. One factor may be affected by another factor that exists simultaneously. It is unclear whether there is a specific factor that plays a key role in the occurrence and development of short stature. Therefore, the correct evaluation and prediction of short stature must be considered from multiple perspectives. Thus, a clinical prediction model for short stature that combines multiple influencing factors is more precise and urgent than focusing solely on a single factor that is highly related to short stature.

At present, there are few studies in the literature on clinical prediction models of short stature; thus, the gap in this area needs to be addressed. This study aimed to establish a clinical prediction model and a risk classification system based on factors that influence short stature and provide additional guidance for clinical screening and prevention of short stature.

## Participants and methods

### Diagnostic criteria and study participants

In this study, we used China's age and sex-specific height growth references for short stature evaluations (19). The diagnostic criteria definded that height was below the third percentile for the mean height of a given age, sex among population group. A total of 15,726 children from 13 primary and secondary schools in Pingshan District, Shenzhen, were selected to participate in a cross-sectional survey through questionnaires. After excluding those with missing data and invalid questionnaires, the participants were included in this study were 12,504. The studies involving human participants were reviewed and approved by the Ethics Committee of the Zhujiang Hospital of Guangzhou City.

### Definition of age

According to the exact number of days from the participant's birth date to the end of the questionnaire, age was calculated as 365 days per year and one digit after the decimal point was retained. The age range was defined as follow: 6–6.9 years was defined as the 6-year-old group, 7–7.9 years was defined as the 7-year-old group, 8–8.9 years was defined as the 8-year-old group, and so forth.

### Questionnaire design

All participants voluntarily completed a questionnaire that included information on the sex, age, date of birth, height, weight, eating habits, behavioral habits, family and social environment, and mother's pregnancy. The height and weight of all subjects were provided by professionals after physical examination and then guardians completed the questionnaire. The BMI in this study was calculated using the weight/height squared (kg/m^2^) based on the height and weight values provided in the questionnaire.

We conducted strict quality control on the questionnaire. We revised the questionnaire according to the feedback from participants and the adoption of expert opinions after the pre-survey stage. During the research process, operators were trained by a professional unified program. Training program included standardized measurement of height and weight, data recording and data computerial input. Unified measuring instrument were used during the whole study. Also, all teachers were trained on how to fill in the questionnaire effectively. Teachers are responsible for sending electronic questionnaires to all parents or guardians of participating children. The data is exported from the electronic questionnaire and transfer into excel version, which is strictly monitored by the researches. In case of missing or uncertain records, parents were contacted to ensure the accuracy of questionnaire data.

### Propensity score matching

This study was a cross-sectional survey study and not a randomized controlled trial; therefore, a selection bias is inevitable. Traditional 1 : 1 propensity score matching (PSM) was introduced to minimize the effect of bias, which could affect the analysis of risk factors in children of short stature. PSM analysis was performed using SPSS version 25.0 (IBM Corp., Armonk, NY, United States). The set-matching tolerance was 0.005, and the number of seeds was 123456. The PSM analysis was adjusted for age.

### Statistical analyses

**Figure 1:**
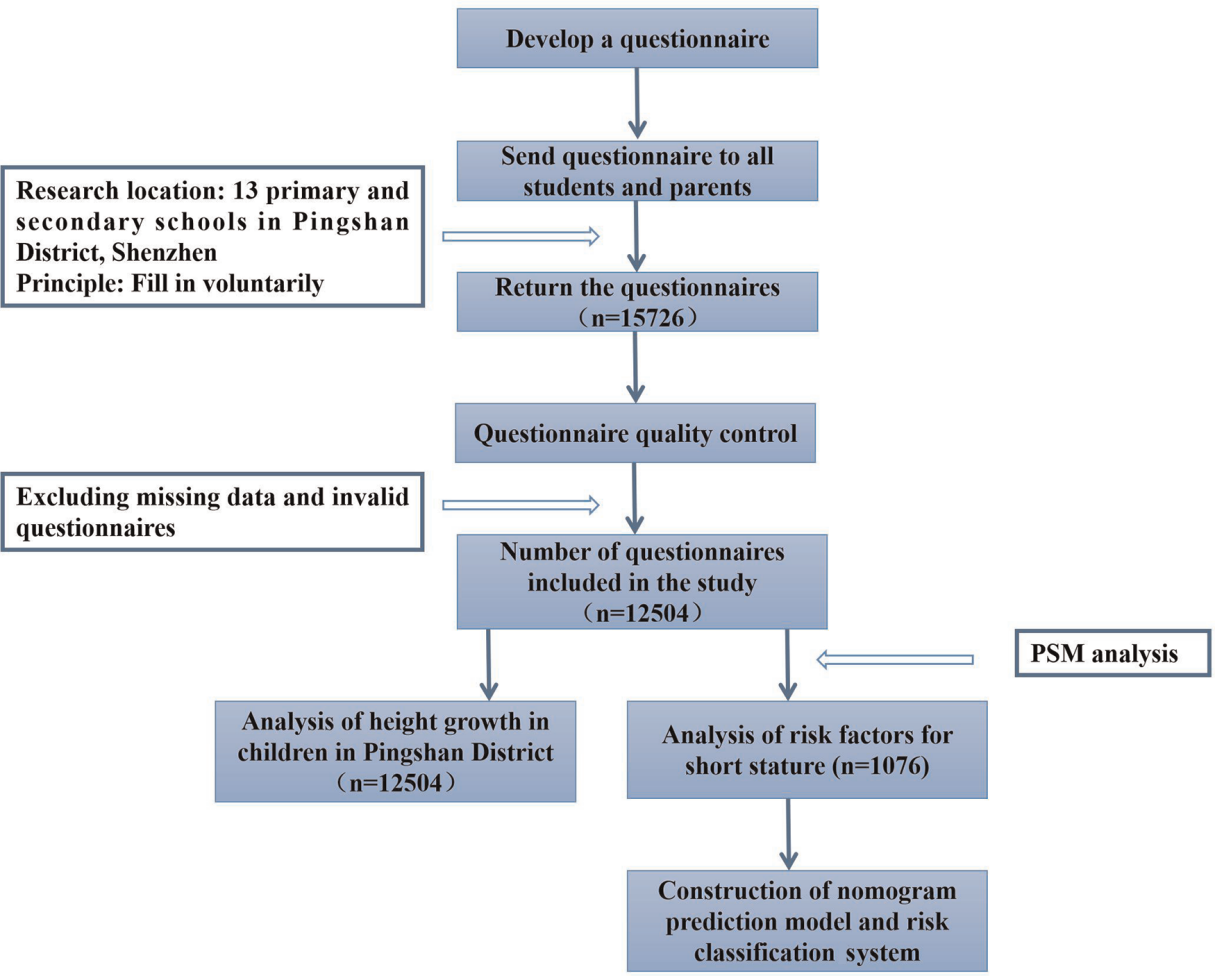
Flow chart of data screening.

Two independent sample *t*-tests were used to compare the heights of different sexes and age groups. Continuous variables were converted to categorical variables. According to the Chinese Adult Obesity Guidelines (20) and 2018 the China Health Industry standard of “Screening for overweight and obesity among school-age children and adolescents” (21), the variable of BMI was adjusted to three categories: normal, overweight, and obese. The chi-square test was used to compare the prevalence of short stature in different sexes and ages. In the PSM-adjusted cohort, the chi-square test was used to compare the differences between the short group and the healthy group in terms of general conditions, family and social environment, mother's pregnancy, childbirth and feeding, eating habits and behavioral habits. The distinct factors in the chi-square analysis were included in the multivariate logistic regression analysis to explore the independent factors influencing short stature. Based on the important influencing factors in the multivariate logistic regression model, a nomogram was established to predict the risk of short stature. The area under the curve (AUC) was used to evaluate the discriminative capacity of the prediction model. The accuracy of the model was evaluated using a calibration curve. Additionally, based on the total score obtained from the nomogram for each patient, patients were divided into high-risk and low-risk groups, and a risk classification system was established. The nomogram and risk classification system are constructed using the software “R studio”. All other statistical analyses were performed using SPSS version 25.0 (IBM Corporation, Armonk, NY, United States). During the entire data analysis process of this study, a *P*-value of <0.05 was considered statistically significant. Figure 1 shows the flow chart for the selection of participants in this study.

## Results

### Population characteristics

**Figure 2:**
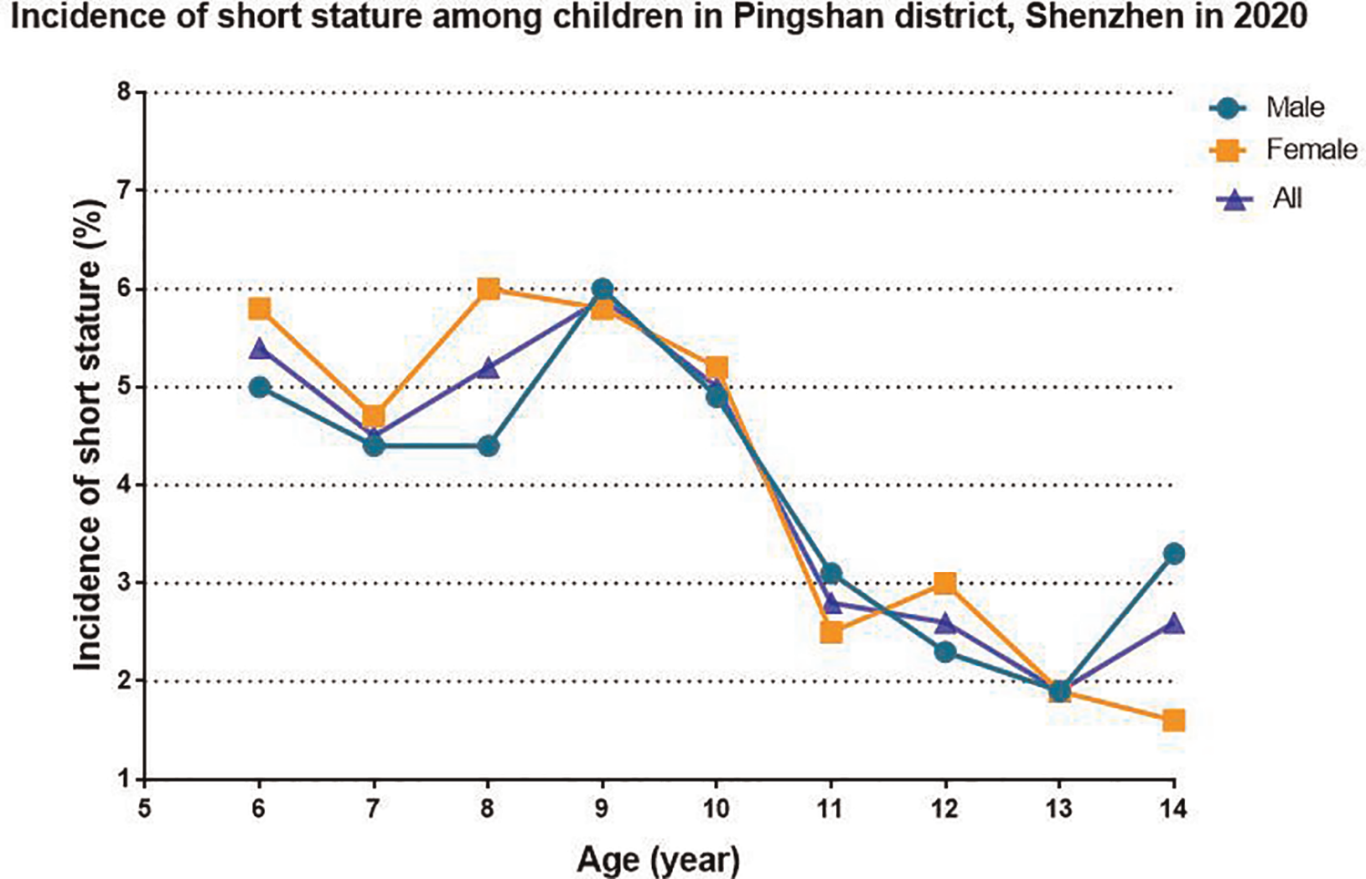
Incidence of short stature among children in Pingshan district, Shenzhen in 2020.

In total, 12,504 valid questionnaires were included in this study. The basic distribution of the samples according to age and sex is shown in Supplementary Table S1. The height distribution characteristics of different sexes among children aged 6–14 are shown in Supplementary Figure S1. The incidence of short stature in children aged 6–14 years was 4.3%. They were 4.1% and 4.5% for boys and girls, respectively. The incidence peaked at the age of 9 in boys and the age of 8 in girls. Then they gradually decreased with age. However, the incidence respectively rose again at the age of 14 and 12 in boys and girls (Table 1, Figure 2).

**Table 1:** Incidence of short stature among children of different genders and ages in Pingshan district, Shenzhen in 2020.

Age (year)	Male (%)	Female (%)	Total	*P* value
6	42/836 (5.0%)	41/701 (5.8%)	83/1,537 (5.4%)	0.476
7	54/1,228 (4.4%)	46/975 (4.7%)	100/2,203 (4.5%)	0.720
8	47/1,057 (4.4%)	54/896 (6.0%)	101/1,953 (5.2%)	0.116
9	51/855 (6.0%)	40/691 (5.8%)	91/1,546 (5.9%)	0.884
10	36/735 (4.9%)	28/537 (5.2%)	64/1,272 (5.0%)	0.799
11	21/675 (3.1%)	13/519 (2.5%)	34/1,194 (2.8%)	0.532
12	17/748 (2.3%)	18/606 (3.0%)	35/1354 (2.6%)	0.421
13	10/531 (1.9%)	9/485 (1.9%)	19/1,016 (1.9%)	0.974
14	8/241 (3.3%)	3/188 (1.6%)	11/429 (2.6%)	0.262
Total	286/6,906 (4.1%)	252/5,598 (4.5%)	538/12,504 (4.3%)	0.324
*P* value	0.001	<0.001	<0.001	

### Matched cohort analysis and risk factors for short stature

The survey population was divided into a short stature group (*n* = 538) and a healthy group (*n* = 11,966) according to their height. The 1 : 1 matching principle was used to match the short stature group and the control group. Age was used as the adjustment variable. The final sample size was 1,076 cases and the short stature and control groups included 538 cases, respectively (Supplementary Figure S2).

### Analysis of influencing factors of short stature

In the PSM-matched cohort, chi-square analysis showed that 11 factors were significantly associated with the risk of short stature in children, of which 8 were negatively correlated, including frequency of physical exercise/week, bowel frequency/week, father's education, mother's education, annual family income, father's BMI, father's height, and mother's height. Two of these factors are positively correlated with the risk of short stature in children: high pressure in life or study and parents’ concerns about children's height. Furthermore, in terms of daily diet preference, children who ate a balanced diet had a lower incidence of short stature (Tables 2, 3). Subsequently, all statistically significant factors were included in the multivariate logistic regression analysis.

**Table 2:** Chi-square analysis between short stature and variables related children, *n* = 1,076.

Variables related children	Short stature (%)	Health (%)	*P* value
Sex	Male	286 (48.9)	299 (51.1)	0.426
	Female	252 (51.3)	239 (48.7)	
Age (year)	6	83 (50.0)	83 (50.0)	0.286
	7	100 (50.0)	100 (50.0)	
	8	101 (49.3)	104 (50.7)	
	9	91 (54.2)	77 (45.8)	
	10	64 (54.7)	53 (45.3)	
	11	34 (36.2)	60 (63.8)	
	12	35 (50.7)	34 (49.3)	
	13	19 (54.3)	16 (45.7)	
	14	11 (50.0)	11 (50.0)	
BMI (kg/m^2^)	Normal	372 (50.5)	364 (49.5)	0.140
	Overweight	64 (43.0)	85 (57.0)	
	Obesity	102 (53.4)	89 (46.6)	
Birth weight	≤4 kg	383 (50.3)	378 (49.7)	0.148
	>4 kg	155 (49.2)	160 (50.8)	
Way of birth	Normal delivery	376 (51.6)	352 (48.4)	0.118
	Cesarean section	162 (46.6)	186 (53.4)	
Infant feeding method	Breast milk	297 (52.5)	269 (47.5)	0.232
	Milk powder	90 (47.1)	101 (52.9)	
	Mixed feeding	151 (47.3)	168 (52.7)	
Daily diet preference	Non-meat	183 (57.7)	134 (42.3)	0.003
	Meat	136 (48.7)	143 (51.3)	
	All	219 (45.6)	261 (54.4)	
Favorite type of drink	Coke	124 (52.5)	112 (47.5)	0.221
	Milk tea	122 (51.3)	116 (48.7)	
	Juice	208 (51.0)	200 (49.0)	
	Others	84 (43.3)	110 (56.7)	
Whether to eat breakfast regularly	Yes	494 (50.1)	492 (49.9)	0.826
	No	44 (48.9)	46 (51.1)	
Frequency of intake of high-calorie foods/week_a_	≤4 times	509 (49.9)	512 (50.1)	0.678
	>4 times	29 (52.7)	26 (47.3)	
Frequency of intake of fried foods/week_b_	≤4 times	511 (50.1)	508 (49.9)	0.683
	>4 times	27 (47.4)	30 (52.6)	
Frequency of eating out and going to restaurants/week	<3 times	489 (50.7)	475 (49.3)	0.458
	3–5 times	23 (40.4)	34 (59.6)	
	5–8 times	11 (50.0)	11 (50.0)	
	>8 times	15 (45.5)	18 (54.5)	
Average time/eating a meal	<15 min	116 (48.9)	121 (51.1)	0.713
	≥15 min	422 (50.3)	417 (49.7)	
Frequency of physical exercise/week	0–2 times	316 (54.4)	265 (45.6)	0.015
	3–4 times	153 (46.1)	179 (53.9)	
	5–6 times	46 (43.0)	61 (57.0)	
	≥7 times	23 (41.1)	33 (58.9)	
Average time of exercising/day	<15 min	151 (53.7)	130 (46.3)	0.105
	15–30 min	266 (51.6)	250 (48.4)	
	30–60 min	92 (43.2)	121 (56.8)	
	60–90 min	23 (46.9)	26 (53.1)	
	>90 min	6 (35.3)	11 (64.7)	
Sleep time/day	3–5 h	2 (40.0)	3 (60.0)	0.446
	5–7 h	53 (55.2)	43 (44.8)	
	7–9 h	401 (50.1)	399 (49.9)	
	9–11 h	82 (47.4)	91 (52.6)	
	>11 h	0 (0.0)	2 (100)	
Sedentary behavior	Yes	106 (50.2)	105 (49.8)	0.939
	No	432 (49.9)	433 (50.1)	
Average time of using electronic media to entertainment	≤60 min	471 (51.4)	446 (48.6)	0.065
	60–120 min	62 (43.4)	81 (56.6)	
	>120 min	5 (31.3)	11 (68.8)	
High pressure in life or study	Yes	177 (57.5)	131 (42.5)	0.002
	No	361 (47.0)	407 (53.0)	
Bowel movement frequency/week	0–2 times	49 (59.8)	33 (40.2)	0.030
	3–4 times	166 (54.2)	140 (45.8)	
	5–6 times	221 (48.4)	236 (51.6)	
	≥7 times	102 (44.2)	129 (55.8)	

a: high-calorie foods mainly includes ice cream, milk tea, chocolate and cake; b: fried foods mainly includes barbecue, french fries, fried chicken and hamburger.

**Table 3:** Chi-square analysis between short stature and variables related parents and family, *n* = 1,076.

Variables related parents and family	Short stature (%)	Health (%)	*P* value
Father's education	College and below	475 (54.6)	395 (45.4)	<0.001
	Bachelor and above	63 (30.6)	143 (69.4)	
Mother's education	College and below	489 (53.7)	421 (46.3)	<0.001
	Bachelor and above	49 (29.5)	117 (70.5)	
Annual family income	<￥100,000	311 (58.0)	225 (42.0)	<0.001
	￥100,000–￥200,000	161 (54.4)	135 (45.6)	
	￥200,000–￥500,000	54 (27.7)	141 (72.3)	
	>￥500,000	12 (24.5)	37 (75.5)	
Father's BMI	Normal	332 (50.3)	295 (49.7)	0.011
	Overweight	173 (48.2)	186 (51.8)	
	Obesity	33 (36.7)	57 (63.3)	
Mother's BMI	Normal	443 (50.3)	438 (49.7)	0.332
	Overweight	80 (51.3)	76 (48.7)	
	Obesity	15 (38.5)	24 (61.5)	
Father's height	<160 cm	21 (91.3)	2 (8.7)	<0.001
	160 cm–170 cm	305 (61.2)	193 (38.8)	
	170 cm–180 cm	206 (39.8)	312 (60.2)	
	≥180 cm	6 (16.2)	31 (83.8)	
Mother's height	<150 cm	23 (88.5)	3 (11.5)	<0.001
	150 cm–160 cm	381 (59.3)	261 (40.7)	
	160 cm–170 cm	131 (33.0)	266 (67.0)	
	≥170 cm	3 (27.3)	8 (72.7)	
Family history of chronic diseases	Yes	57 (43.5)	74 (56.5)	0.113
	No	481 (50.9)	464 (49.1)	
Parents’ worries about children's height	Yes	358 (62.2)	218 (37.8)	<0.001
	No	180 (36.0)	320 (64.0)	
History of maternal gestational diabetes or hypertension	Yes	9 (37.5)	15 (62.5)	0.421
	No	518 (50.4)	510 (49.6)	
	Unknown	11 (45.8)	13 (54.2)	
History of mother smoking during pregnancy	Yes	1 (33.3)	2 (66.7)	0.563
	No	537 (50.0)	536 (50.0)	

**Figure 3:**
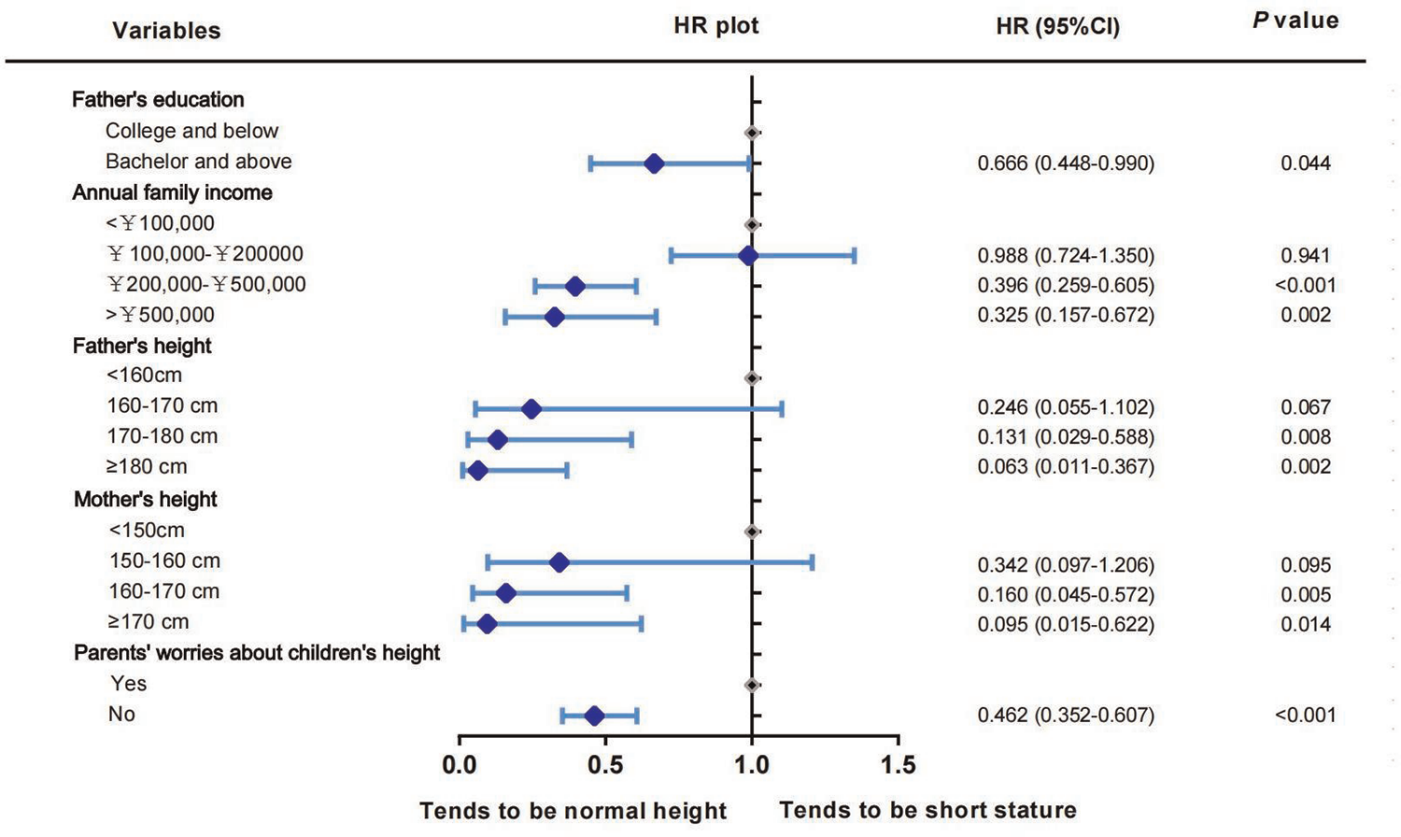
Multivariate logistic regression forest plot of short stature in children.

The multivariate regression analysis revealed predictors of short stature (Figure 3). Five factors were significantly associated with the risk of developing short stature in children, including father's education, annual family income, father's height, mother's height, and parents’ concerns about children's height. Factors that helped reduce the risk of short stature were father's education, annual family income, father's height, and mother's height. Conversely, another factor was the parents’ concern about their children's height. Parents became more concerned about their children's future height, which was associated with a higher risk of short stature. These five factors were selected as significant predictors of short stature in children.

### Construction of a nomogram prediction model and risk classification system

**Figure 4:**
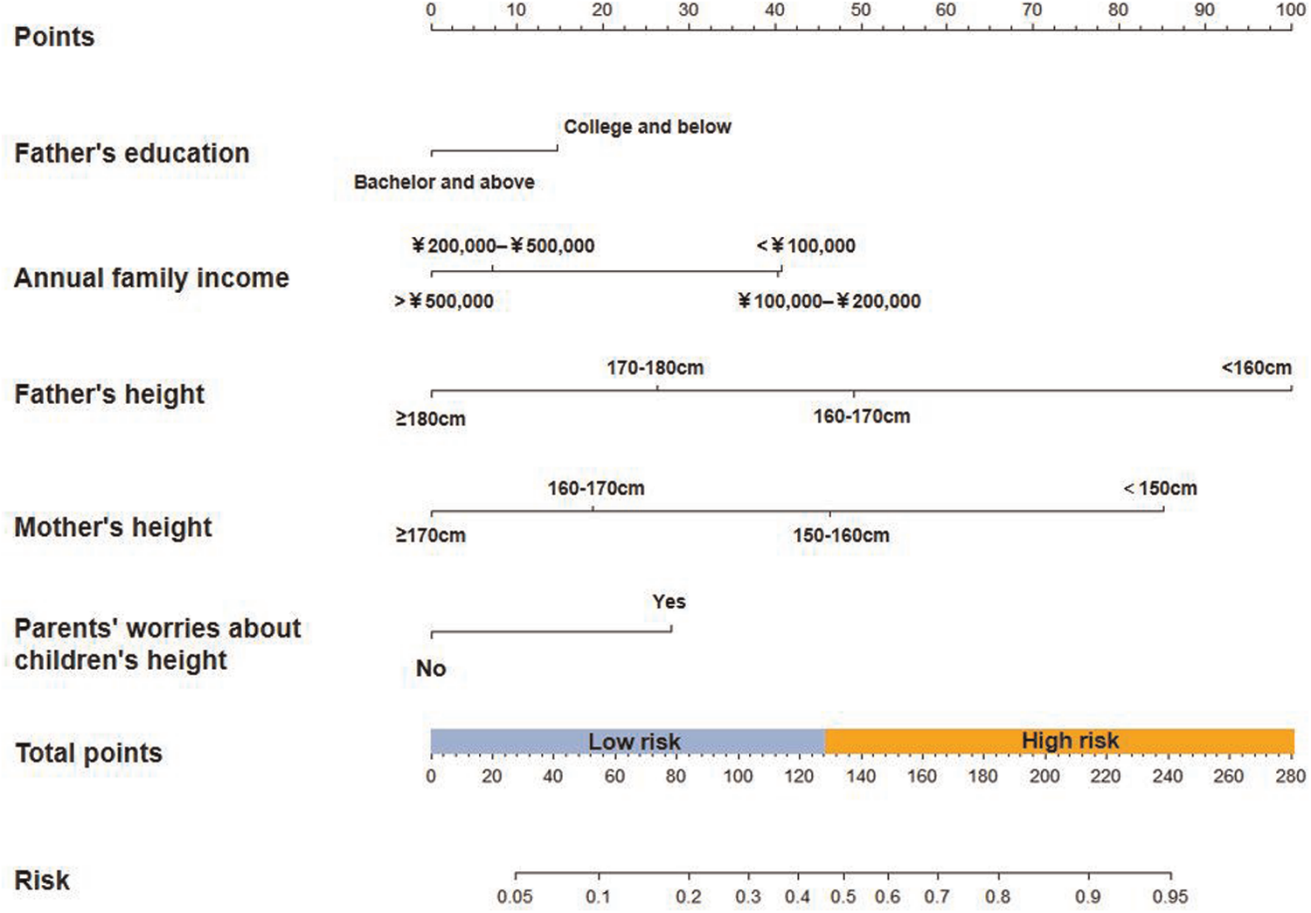
The prediction nomogram models and risk classification system for short stature in children.

**Figure 5:**
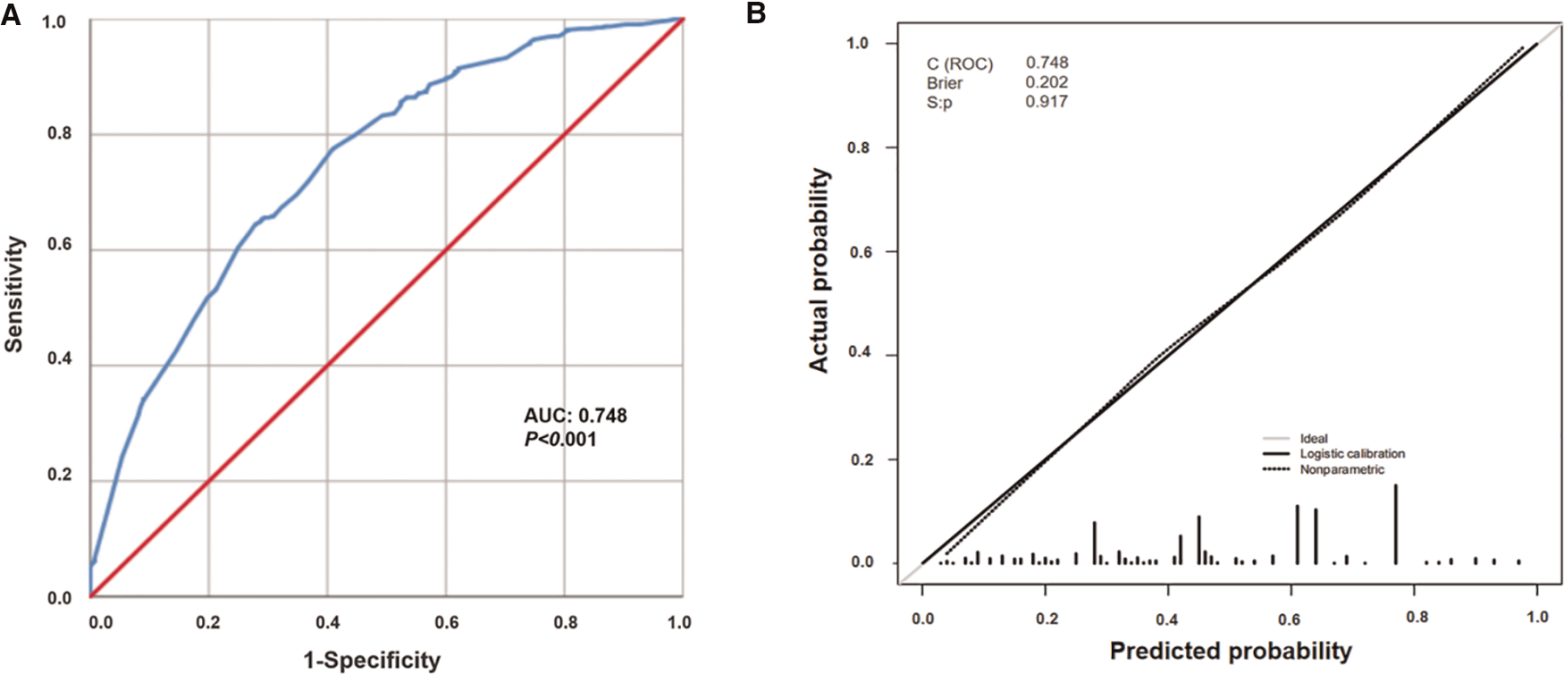
Evaluation of nomogram prediction models. (**A**) Area under the ROC of the nomogram model; (**B**) the calibration curve of the nomogram model. Dashed line represents ideal prediction, and solid line represents observed nomogram.

To visually assess the risk of short stature, a nomogram prediction model was constructed for short stature in children to identify important predictors. From the nomogram, it was found that the height of the parents had the greatest influence on whether the child may have short stature in the future, followed by annual family income and parents’ concerns about children's height. The educational level of the father had the least influence. Based on the results of the nomogram, each factor was scored accordingly to calculate the risk of short stature for each child (Figure 4 and Supplementary Table S2). The discrimination and precision of the nomogram model were evaluated using ROC and calibration curves. The AUC of the model was 0.748 (95% CI: 0.719–0.777), indicating that the nomogram prediction model had a good degree of discrimination. The Hosmer–Lameshaw test showed a *P*-value = 0.917. The prediction model had a good degree of calibration and the actual probability of short stature was in good agreement with the predicted probability (Figure 5).

A risk classification system was developed based on the total nomogram score for each child. The ROC curve was used to determine a cutoff value of 127.5, with which all children were divided into two risk groups: a low-risk group (439/1,076, 40.80%; score 0–127.5) and a high-risk group (637/1,076, 59.20%; score 127.5–269) (Figure 4).

## Discussion

Short stature has become an important public health issue that affects approximately 10% of children worldwide (22). This study selected 12,504 children as research subjects in the Pingshan District, Shenzhen, China. The purpose of this study was to understand the incidence of short stature, analyze risk factors, and build a nomogram model and risk classification system for short stature. The constructed nomogram visualizes risk factors and can predict the probability of risk and the degree of short stature in children.

Regarding the growth of height, the heights of boys and girls showed stage differences in age distribution, which may be related to factors such as the onset of puberty and the age of menarche. Puberty was affected by many factors such as sex hormones. Therefore, physical growth should have a second peak after birth. However, gender differences in height would be reflected after this period. According to the previous researches which in the field of child growth and development (23–26), in well-nourished populations, the timing of peak height velocity occured around age 11 years in girls (±2 SD about range 9–14 years) and 12–13 years in boys (±2 SD about range10–14 years). In our study, we found the incidence in boys peaked at the age of 9 and the incidence of girls peaked at the age of 8, then the incidence of all almost declined over time. This result was consistent with the growth and development time of children. The overall prevalence of short stature in children in this study was 4.3%, corresponding to 4.1% among boys and 4.5% among girls. However, there were no significant differences in the prevalence of short stature between boys and girls, which is consistent with the findings of a study conducted in Saudi Arabia (27). Wang et al. found that the average rate for short stature among primary and secondary school students in Anhui Province was 3.16% in 2015 (3). A survey of 213,795 Han children found that the prevalence of short stature among children aged 7–18 years was 3.70% in 30 provinces of China in 2014 and suggested that the prevalence of short stature was higher in southwest of China but lower in northeast China (2). The factors that cause the differences in the prevalence of short stature among children in different regions are not yet clear, but may be closely related to factors such as heredity, region, race, gender, nutrition, various endocrine hormones, economic level, and living conditions. Furthermore, compared to other studies that adopted the diagnostic criteria of 2 SD below the mean height, the diagnostic criterium for short stature in this study was the third percentile below the mean height, leading to a high incidence of short stature (28).

This study also identified predictors of short stature. The nomogram prediction model showed that parental height significantly affected a child's height. Height growth in children is undoubtedly a multifactorial process that involves genetic and environmental factors. A study that analyzed the height of 6,752 individuals from 2,508 families determined the heritability of height between 0.75 and 0.98 (27). Genetic factors significantly affect a child's height. The risk of short stature in children decreases with taller parents. Therefore, early screening, dynamic follow-up, and timely intervention should be performed in children with shorter parents. Furthermore, our findings support previous findings (6, 29–31) that environmental factors independently influence the risk of short stature in children. In the nomogram prediction model, both annual family income and fathers’ education level were moderately but significantly associated with the risk of short stature in children. Ghajar et al. (32) evaluated a longitudinal cohort of 10,127 children and found that both parental income and education were associated with higher height-for-age *z*-scores (HAZ), which is consistent with the results of previous studies (33, 34). In our study, these two factors independently influenced children's height. It is speculated that in a family structure, due to the higher education level of fathers, higher family income and superior socioeconomic status are related to the good nutritional status of children. Due to traditional Chinese family values, males have a higher family status, which may cause the father's education level to have a more significant effect on short stature in children than the mother's education level. A study (35) of the cohort who were born in 1958 and their descendants found that environmental factors, such as mother's education and social class, had a smaller effect on the height of the offspring, which is similar to our findings. However, this study found that parents’ concerns about their children's height was a risk factor for short stature. Parents’ awareness of worrying about their children's height was greater and the risk of short stature in their children increased 1.164 times. Psychologists describe this phenomenon as the “focusing illusion” (36). This phenomenon indicates that paying too much attention to a certain factor results in excessive pressure. Stronger worry-conscious parents may be more concerned about their children's height, resulting in ignoring or downplaying other aspects of life that may be psychologically beneficial or harmful to their children. Continuous psychological disturbances can adversely affect children's growth and development. Good attitude and correct communication among parents are important medical behaviors that are beneficial for the growth and development of children.

Previous studies have reported inconsistent results regarding the effect of obesity on height in children (37). Therefore, in the analysis process of this study, the PSM approach was adopted to minimize confounding bias, and the results showed that obesity and lifestyle were not independent risk factors for short stature.

Based on the nomogram prediction model, this study constructed a risk classification system for short stature and divided patients into high-risk and low-risk groups. Clinicians can assess the probability and severity of short stature in each child based on the nomogram scores and the risk classification system, providing individualized treatment and follow-up plans for each child. Also, according to the findings of this study, specific intervention methods should be considered to prevent short stature. Firstly, we need to raise the awareness of short stature for parents even educators and society, including actively conducting health education in the field of growth and development as well as emphasizing harm of short stature. Furthermore, regular height monitoring and height management are very important. If parents are short, low education and income groups, they should pay more attention to the growth and development of their children at an early age. Last but not least, both of children's pressure and parental stress were noticed to reduce by improving individual and family adaptation. Multidisciplinary interventions in pediatric endocrinology should be taken with family-centred.

## Conclusions

Overall, given its good clinical utility and convenience compared to conventional evaluation methods, our nomogram model and risk classification system are effective assessment methods for the early screening of short stature. Furthermore, our findings provide an early intervention strategy for preventing short stature. Regular screening and follow-up of height is more important for children whom with risk factors.

Nevertheless, as a cross-sectional study, it also had some limitations. First, the data were collected through a questionnaire, which may have resulted in a reporting bias, although we have undergone strict quality control. Second, height growth is a complex process affected by many factors, and confounding factors were not considered in this study. For example,the information on medical examination related to short stature including growth hormone, thyroid hormone and bone age, the information about residence and small gestational age of participants. Third, owing to lacking of longitudinal follow-up data in this study, external validation of this predictive system was not possible. We develop a five-year follow-up plan for this population and are already to implement it. External validation of the predictive model will be supplemented while follow-up data enough.

## Data Availability

The raw data supporting the conclusions of this article will be made available by the authors, without undue reservation.
