# Supplementary material for: Analysis of risk factors and construction of a prediction model for short stature in children
Source: Front Pediatr. 2022 Dec 6;10:1006011. doi: 10.3389/fped.2022.1006011 (PMC9763591; doi:10.3389/fped.2022.1006011)
Supplement: Supplementary file 1 [file Datasheet1.pdf]

Supplementary table 1 Univariate logistic regression analysis for variable associated with short stature

Variable		Univariate logistic regression analysis	
		HR	P
Parents' worries about children's future height	Yes	Reference	
	No	0.395(0.221-0.707)	0.002

Supplementary table 2 Chi-square test for variable associated with short stature

Variable		Short stature (%)	Health (%)	*P* value
Parents' worries about children's future height	Yes	31 (39.2)	48 (60.8)	0.001
	No	36 (20.3)	141 (79.7)	

Supplementary table 3 The prevalence of short stature in Pingshan and other cities

Districts	Result source	Prevalence of short stature(%)
Pingshang	This study	4.3
Nationwide prevalence	Ma J et al (2019)	3.67
Beijing		0.62
Tianjin		0.75
Hebei		1.29
Shanxi		3.08
Inner Mongolia		1.90
Liaoning		1.10
Jilin		1.81
Heilongjiang		2.86
Shanghai		0.84
Jiangsu		0.72
Zhejiang		1.30
Anhui		2.10
Fujian		1.13
Jiangxi		2.00
Shangdong		0.42
Henan		1.76
Hubei		2.53
Hunan		4.80
Gansu		3.39
Qinghai		6.20
Ningxia		3.42
Xinjiang		4.11

Adolescent Health Management Questionnaire

1. What school the student attends? (Fill in the blank)

____

2. Student's name (Fill in the blank)

____

3. Student's age ______years old

Student's date of birth ________________ (Fill in the blank)

4. Student's gender (Single Choice)

○Male

○Female

5. Contact details for parents (Telephone number) (Fill in the blank)

____

6. Parents' WeChat ID (Follow-up via WeChat) (Fill in the blank)

____

7. The education level of the student's father (Single Choice)

○Elementary school and below

○Junior high school

○High school or college

○Bachelor

○Master degree and above

8. The education level of the student's mother (Single Choice)

○Elementary school and below

○Junior high school

○High school or college

○Bachelor

○Master degree and above

9. The total annual income of the student’s family is approximately_____ (Single Choice)

○<￥100,000

○￥100,000-￥200,000

○￥200,000-￥500,000

○>￥500,000

10. Student's birth weight is _____ (Single Choice)

○＜4kg

○＞4kg

11. Way of student’s birth (Single Choice)

○Normal delivery

○Cesarean section

12. Infant feeding method (Single Choice)

○Breast milk

○Milk powder

○Mixed feeding

13. Breast milk feeding duration (Single Choice)

○Less than half a year

○Half a year to one year

○More than one year

14. Current student's weight______kg，height ______cm (Fill in the blank)

15. Weight of student's father ______kg，height ______cm (Fill in the blank)

16. Weight of student's mother ______kg，Height ______cm (Fill in the blank)

17. Does the student's family have a family history of chronic diseases，including hypertension, diabetes, hyperlipidemia, cardiovascular and cerebrovascular diseases, etc？ (Single Choice)

○No

○Yes（Please specify who in the family has what disease） _________________ *

18. Whether the student suffers from diabetes, hyperuricemia or gout, hyperlipidemia? (Single Choice)

○No

○Yes（Description of specific diseases） _________________ *

19. Are there any abnormal indications in blood glucose , blood lipids and blood pressure in the student's previous physical examination？ (Single Choice)

○Yes (Please specify for details) _________________ *

○No

20. Did the student's mother have gestational diabetes or gestational hypertension? (Single Choice)

○Yes (Please specify for details) _________________ *

○No

○Unknow

21. Did the student's mother have a history of smoking during pregnancy？ (Single Choice)

○Yes

○No

22. Students' daily diet preference (Single Choice)

○Whole grains

○Vegetables

○Fruit

○Meat

○All

23. What is the student's favorite drink？ (Single Choice)

○Coke

○Milk tea

○Juice

○Others _________________ *

24. Do students eat breakfast regularly? (Single Choice)

○Yes

○No

25. Frequency of student intake of ice cream, milk tea, chocolate or cake (Include one of them) (Single Choice)

○1-2times/Week

○3-4times/Week

○5-6times/Week

○≥7times/Week

○No

26. Frequency of student intake of barbecue, fries, fried chicken, burgers (Include one of them) (Single Choice)

○1-2times/Week

○3-4times/Week

○5-6times/Week

○≥7times/Week

○No

27. Number of times students eat takeaway and eat in restaurants (Single Choice)

○＜3times/week

○3-5times/week

○6-8times/week

○＞8times/week

28. Average meal time for students (Single Choice)

○＜15min

○15-30min

○＞30min

29. The frequency of physical exercise by students (Single Choice)

○1-2times/week

○3-4times/week

○5-6times/week

○≥7times/week

○No

30. Average daily exercise time for students (Single Choice)

○＜15min

○15-30min

○30-60min

○60-90min

○＞90min

31. Daily sleep time of students (Single Choice)

○3-5 hours

○5-7 hours

○7-9 hours

○9-11hours

○＞11hours

32. Whether the student is often sedentary (3 hours of continuous sitting) ? (Single Choice)

○Yes

○No

33. Average daily entertainment time of students using electronic media (Single Choice)

○＜30min

○30-60min

○60-90min

○90-120min

○＞120min

34. Whether the students often feel intense stress in life or study? (Single Choice)

○Yes

○No

35. Student's weekly bowel movements (Single Choice)

○0-2 times/Week

○3-4 times/Week

○5-6 times/Week

○≥7 times/Week

36. Parents' worry about their child's weight in the future (Single Choice)

○Yes

○No

37. Parents' worry about their child's height in the future (Single Choice)

○Yes

○No

38. Would parents be willing to learn about measures to prevent obesity or diabetes? (Single Choice)

○Yes

○No
