# Supplementary material for: Analysis of risk factors and construction of a prediction model for short stature in children
Source: Front Pediatr. 2022 Dec 6;10:1006011. doi: 10.3389/fped.2022.1006011 (PMC9763591; doi:10.3389/fped.2022.1006011)
Supplement: Supplementary file 2 [file Datasheet2.docx]

Supplementary Material

# Supplementary Figures and Tables

## Supplementary Figures


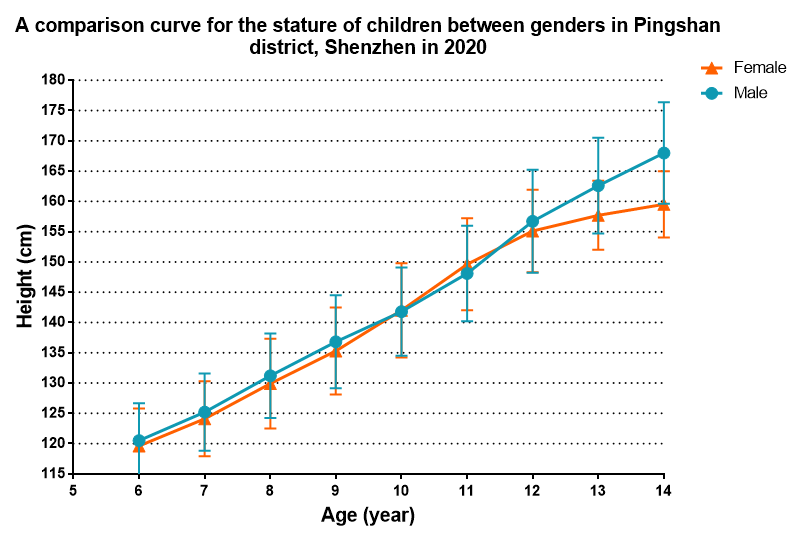


1. A

**Supplementary Figure 1.** A comparison curve for the stature of children between genders


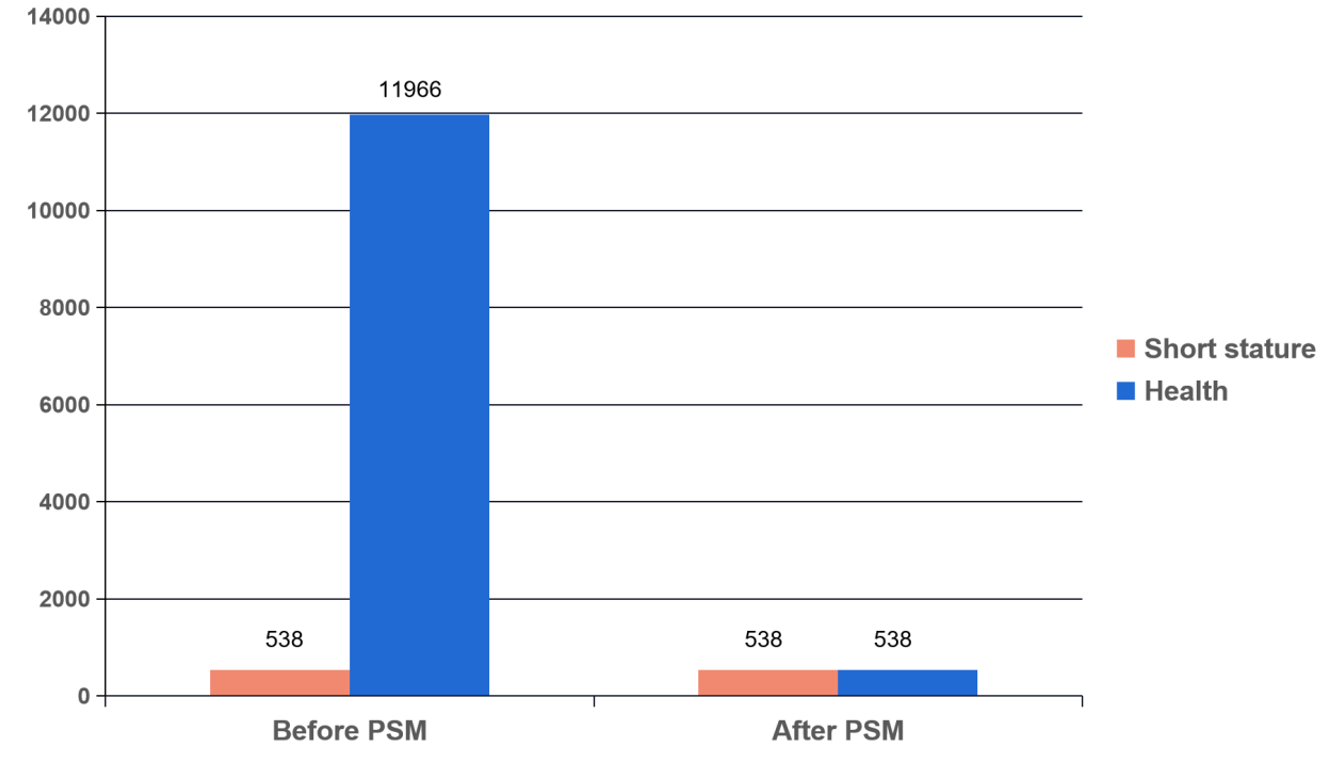


**Supplementary Figure 2.** Sample distribution before and after propensity score matching analysis

## Supplementary tables

**Supplementary table 1.** Sample distribution of children in Pingshan district, Shenzhen

| 年龄（岁）  性别 | 男（%） | 女（%） | 合计 |
| --- | --- | --- | --- |
| 6 | 836（54.4%） | 701（45.6%） | 1537 |
| 7 | 1228（55.7%） | 975（44.3%） | 2203 |
| 8 | 1057（54.1%） | 896（45.9%） | 1953 |
| 9 | 855（55.3%） | 691（44.7%） | 1546 |
| 10 | 735（57.8%） | 537（42.2%） | 1272 |
| 11 | 675（56.5%） | 519（43.5%） | 1194 |
| 12 | 748（55.2%） | 606（44.8%） | 1354 |
| 13 | 531（52.3%） | 485（47.7%） | 1016 |
| 14 | 241（56.2%） | 188（43.8%） | 429 |
| 合计 | 6906（55.2%） | 5598（44.8%） | 12504 |

|  | Variables | Score |
| --- | --- | --- |
| Father's education | High school or junior college and below | 15 |
|  | University and above | 0 |
| Annual family income | <100,000 yuan | 41 |
|  | 100,000-200,000 yuan | 40 |
|  | 200,000-500,000 yuan | 7 |
|  | >500,000 yuan | 0 |
| Father's height | ＜160 cm | 100 |
|  | 160-170 cm | 49 |
|  | 170-180 cm | 26 |
|  | ≥180 cm | 0 |
| Mother's height | ＜150 cm | 85 |
|  | 150-160 cm | 46 |
|  | 160-170 cm | 19 |
|  | ≥170 cm | 0 |
| Parents' awareness of worrying about children's future height | Yes | 28 |
|  | No | 0 |

**Supplementary table 2.** Scores of predictors in nomogram model of short stature in children
